# Effects of sitting on the respiratory pattern, mechanics and work of breathing in mechanically ventilated patients

**DOI:** 10.1186/cc13449

**Published:** 2014-03-17

**Authors:** F Ruiz-Ferron, J Serrano-Simon

**Affiliations:** 1 Complejo Hospitalario de Jaen, Spain; 2 Hospital Reina Sofia, Cordoba, Spain

## Introduction

The effect of sitting in an armchair on mechanically ventilated patients has not been studied enough. We study a group of patients ready for weaning for the respiratory pattern, mechanics and work of breathing during reclining in bed and after sitting in an armchair.

## Methods

Thirteen patients who needed mechanical ventilation after 18 days (1 to 60) were studied during volume assist-control mechanical ventilation and spontaneous breathing (O_2_T, CPAP or PSV) in both positions. Airways, esophageal pressures and flow were registered for posterior analysis. Passive respiratory mechanics were measured by multiple linear regression methods, respiratory drive in esophageal pressure (P01) and respiratory effort using the pressure time product (PTP).

## Results

On controlled mechanical ventilation the respiratory system and chest wall elastance were significantly higher in sitting compared with reclining positions (Ers 39 ± 24 vs. 33 ± 25 cmH_2_O/l and 10 ± 3 vs. 7 ± 3 cmH_2_O/l), respiratory resistances were similar (15 ± 3 vs.14 ± 5 cmH_2_O/l/second) in both positions. Breathing pattern did not change significantly: tidal volume (0.406 ± 0.108 vs. 0.394 ± 0.118 l), inspiratory flow (0.74 ± 0.27 vs. 0.69 ± 0.23 l/second), inspiratory (0.97 ± 0.23 vs. 0.97 ± 0.28 seconds) and expiratory (1.43 ± 0.55 vs. 1.39 ± 0.44 seconds) times and respiratory frequency (27 ± 7 vs. 26 ± 7 bpm). Respiratory drive and effort tend to be higher in the sitting position, but not significantly. P01: 3.7 ± 1.9 versus 3.0 ± 1.4 cmH_2_O, Apes: 19 ± 10 versus 15 ± 8 cmH_2_O, PTPmin: 373 ± 192 versus 284 ± 162 cmH_2_O/second*minute.

## Conclusion

A sitting position for mechanically ventilated patients increased the rigidity of chest wall and the respiratory system. The effects of this mobilization must be evaluated because some patients show higher respiratory drive and effort in this position.

